# General Francis Amasa Walker

**Published:** 1897-02

**Authors:** 


### Obituary.

GENERAL FRANCIS AMASA WALKER, A. M., L. L. D., president of the Massachusetts Institute of Technology, died at Boston, January 5, 1897, aged 56 years. He was in good health up to the time of retiring on the evening of the 4th, but just before midnight he was stricken with apoplexy. Mrs. Walker was aroused by his hard breathing and sent for a physician, but the general died before medical aid reached his bedside. Besides the widow, General Walker leaves five children, three sons and two daughters.

---

General Walker was born at Boston, July 2, 1840. He was the son of Amasa Walker, the eminent educator and political economist, and himself obtained conspicuous eminence in these fields. He graduated from Amherst in 1860, and then studied law, but entered the union army in 1861. In 1862, he became assistant adjutant-general on the staff of General Sumner, commanding the second army corps, on which duty he soon rose to the rank of Lieut.-colonel. He served in this capacity with the several commanders of the second army corps—Sumner, Couch, Hancock, Humphries—until his retirement from the service in 1865, at which time he was commissioned as brevet brigadier-general of volunteers for gallant service. General Walker was severely wounded in his left hand at Chancellorsville, from the effects of which he barely escaped lock-jaw, while his hand was ever after deformed and nearly incapable of prehensile power. After the war he performed editorial work on the *Springfield Republican*, and in 1869 took charge of the Bureau of Statistics at Washington.

In 1870, he was appointed superintendent of the ninth census, and for a time also performed the duties of Indian Commissioner during a temporary vacancy in that office in 1871. In 1873, he was appointed professor of political economy at the Sheffield Scientific School, and in 1881 was elected president of the Massachusetts Institute of Technology, which office he held from that time forward until his death. He also served as chief of awards at the centennial exposition in 1876, and was one of the United States commissioners at the international money conference in 1878. He was also superintendent of the tenth census, 1880-81.

General Walker was best known as a student of political economy, in which department of science he was recognised as authority and his treatise on that subject is in general use as a text-book. He was a strong advocate of international bimetalism from a scientific standpoint. General Walker's writings include his Political Economy, Money and its relation to trade and industry, The wages question, Land and its rent, (a reply to Henry George's Progress and Poverty), Money and the Indian question. He also wrote the History of the Second Army Corps, was a frequent contributor to the *Atlantic Monthly* and other magazines, and also contributed editorials to many of the leading newspapers. His statistical atlas of the United States and his work on the ninth and tenth censuses have established his fame, far and wide, as an accurate statistician. General Walker was a companion of the Military Order of the Loyal Legion, and at one time commander of the Massachusetts Commandery.

---

General Walker's funeral was held at Trinity Church, Boston, at noon, Friday, January 8, 1897, at which were gathered distinguished citizens and intimate friends from all parts of the country. The demand for admittance was so great that the capacity of the church proved inadequate to accommodate the thousands who desired to attend the obsequies. Tickets were therefore limited to those who by reason of their official position or intimate association with the great man, whose memory they desired to honor, had claims that could not be overlooked.

Even the pupils from the Institute of Technology were limited to 100 members from each class and they filled the entire gallery.

Delegations were present from the National Academy of Sciences, Harvard College, the Worcester Polytechnic Institute, the Institute of Fine Arts, the public library, the Military Order of the Loyal Legion, the St. Botolph Club, the Commercial Club, the Delta Kappa Epsilon Fraternity, the American Historical Association, the American Statistical Association, Wellesley College, the English Bimetallic League, the Round Table Club, the Municipal League of Boston, the Board of Aldermen and Common Council.

Although in deference to the wishes of the family there was no suggestion of a military funeral, 1200 students of the Institute of Technology escorted the remains from the residence, on Beacon street, to the Church, with the senior students at their head.

Among the honorary pall-bearers were :

Governor Wolcott, Mayor Quincy, Professor Asaph Hall, of the National Academy of Sciences ; Hon. William Endicott, Jr. ; President Merrill E. Gates, of Amherst College ; Dr. George J. Brush, Yale University ; President Eliot, of Harvard University, and Colonel John S. Hazard, of the Military Order of the Loyal Legion.

Rev. E. Winchester Donald and Rev. William Dewart officiated.

The casket was borne down the aisle by student bearers and the honorary pall-bearers. It was covered with wreaths and flowers. The Episcopal service for the dead, without eulogy, was the extent of the ceremony.

It was an impressive sight as the choir sang O, Paradise, and the vast congregation rose while the body was borne from the church, escorted by Governor Wolcott, Mayor Quincy and the other honorary pall-bearers. Outside the church there stood many people with uncovered heads as the funeral cortege drove away to the last resting place at Mount Auburn.

---

The death of General Walker is more than an ordinary loss. His field of usefulness was wide, his judgment was mature and his opinions valuable, and he was the type of a man that can illy be spared at this juncture. His talents were such as to peculiarly fit him to aid in the solution of monetary and economic problems. It was the good fortune of the writer to know him well—years ago even intimately—and he esteems it a duty, be it never so sad, to place on record this slight tribute to his old companion in arms and comrade of the second army corps.

